# Quality of Care Transition During Hospital Discharge, Patient Safety, and Weight Regain After Bariatric Surgery: a Cross-Sectional Study

**DOI:** 10.1007/s11695-023-06486-6

**Published:** 2023-02-11

**Authors:** Matthias Marsall, Alexander Bäuerle, Till Hasenberg, Laura Schräpler, Anita Robitzsch, Marco Niedergethmann, Martin Teufel, Matthias Weigl

**Affiliations:** 1 Institute for Patient Safety (IfPS), University Hospital Bonn, 53127 Bonn, Germany; 2 Clinic for Psychosomatic Medicine and Psychotherapy, LVR-University Hospital Essen, University of Duisburg-Essen, 45147 Essen, Germany; 3 Helios Obesity Center West, Helios St. Elisabeth Hospital Oberhausen, Witten/Herdecke University, Helios University Hospital Wuppertal, 42283 Wuppertal, Germany; 4 Department of Surgery, Obesity and Metabolic Surgery Center, Alfried Krupp Hospital Essen, 45131 Essen, Germany

**Keywords:** %TWL, Patient safety, Care transition, Discharge management, Patient empowerment, Bariatric surgery, Moderation analysis, Interaction effect

## Abstract

**Purpose:**

Bariatric surgery is established as the gold standard in the treatment of severe obesity. However, a significant proportion of patients experience a substantial weight regain afterwards. Previous research focused predominantly on patients’ personal factors. Yet, critical discharge process factors that contribute to patient’s adherence after surgical interventions are rarely examined. This study investigated whether high quality of care transitions in discharge management influences weight regain and the likelihood of experiencing adverse patient safety incidents.

**Materials and Methods:**

A cross-sectional study with 578 patients after bariatric surgery was conducted. Participants answered a standardized assessment on the quality of care transition from hospital to home-, surgery-, and nutrition-related characteristics as well as patient safety incidents.

**Results:**

Significant weight regain was observed 24 months after surgery. The association between time since surgery and weight regain was weaker in patients with high quality of care transitions (*B* = 2.27, *p* < .001). Higher quality of care transition was also significantly related to a lower likelihood of unplanned hospital readmissions (OR = 0.67) and fewer medication complications (OR = 0.48) after surgery.

**Conclusion:**

This study sheds first light on the key influence of high quality of care transitions after bariatric surgery. Improvement efforts into effective discharge processes may establish smoother care transitions and help patients to assume responsibility and compliance with behavioral recommendations after surgery. Moreover, adverse patient safety incidents are less frequent after high quality care transitions indicating both high quality of health services for patients and reducing costs for the health care system.

**Graphical Abstract:**

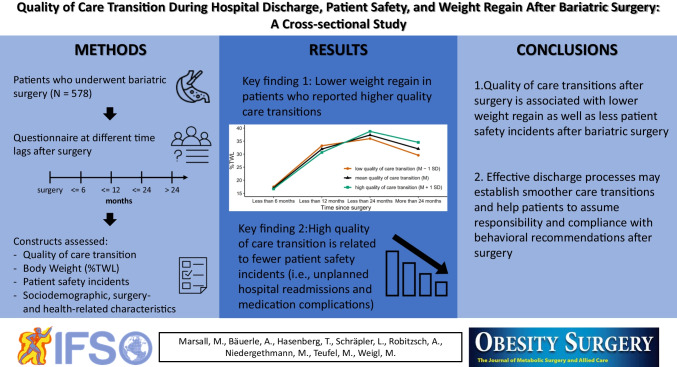

## Introduction

Bariatric surgery is one of the most efficient weight loss treatments for people with obesity [1, 2]. However, weight regain of patients after bariatric surgery is a well-known phenomenon and challenging condition for patients, providers, and the healthcare system [3–5]. Weight regain is a common indicator of the long-term treatment success with an incidence of about 20–25% after bariatric surgery [6]. Studies show that unintentional increase in body weight usually occurs about 18 to 24 months after bariatric surgery [3]. Several factors influencing this issue were examined with the result that mostly patients’ personal factors such as higher preoperative body mass index (BMI), inappropriate postoperative nutrition behaviors, or lack of physical activity are drivers of this challenging condition [2, 6–8]. On the other hand, studies examined several contributing factors to mitigate weight regain with particular relevance of physical activity [9, 10] and adherence to nutrition guidelines [11].

Nevertheless, weight regain is still a key challenge for patients after bariatric surgery [7] with frequent adverse consequences, especially for patients’ mental health [12]. Usually, pre- and post-surgery factors are identified as possible drivers of weight regain [13, 14]. However, studies that focus specifically on organizational factors of intra-hospital processes are currently missing in bariatric surgery research [6]. Research of one organizational factor, the quality of care transitions in the course of patients’ discharge, has shown that a high quality of care transition is associated with better health outcomes and fewer unplanned hospital readmissions [15–17]. High quality of care transition is characterized by effective information transfer and patients’ preparations to promote empowerment and self-management [18, 19]. Empowerment of patients in their journey throughout their in-hospital care is shown to be a key driver to ensure high-quality care [20], patient safety [21], and medication adherence [22]. Moreover, patient empowerment is associated with increased health-related locus of control and self-efficacy [23], as well as higher adherence to health-beneficial behaviors [24]. Eventually, patients after bariatric surgery benefit from higher self-efficacy as mitigating factors of weight regain [25, 26].

Although there is a strong emphasis on high quality of care transitions as an organizational factor to foster patient empowerment, the empirical evidence base in bariatric surgery patients is limited and inconsistent. Systematic investigations into the role of discharge management as an engaging process to support bariatric surgery patients’ self-management competencies are missing. Moreover, potential ramifications of quality of care transitions beyond patient’s weight trajectories are lacking (i.e., safety endpoints). Insights into effects of care transitions after bariatric surgery may inform future improvement efforts to design patient-oriented care services that foster improved long-term surgical outcomes.

Therefore, this study aimed to investigate two questions. Firstly, we aimed to examine the role of quality of care transitions in patients’ weight regain after bariatric surgery. Especially, we were interested whether the effect of time lag since surgery on patients’ weight regain is weaker in patients with higher quality of care transitions. Secondly, we evaluated whether a higher quality of care transition is associated with reduced likelihood of adverse patient safety incidents after bariatric surgery, i.e., unplanned hospital readmissions and medication complications.

## Methods

### Participants and Procedures

We conducted a cross-sectional study between March and May 2022. The data were collected via Unipark (Tivian XI GmbH) platform. We recruited patients at the Obesity and Metabolic Surgery Center of Alfried Krupp Hospital (Essen, Germany), which is a board-certified reference center of care. Further, we addressed patients via an online flyer containing information on the study to recruit participants in topic-related social media groups. Eligibility criteria were age over 18 years, being fluent in German, and undergone bariatric surgery (primary or revisional) in the past. In total,* N* = 616 patients responded to our assessment. Electronic informed consent was obtained from all participants before the survey was shown. The participation in this study was anonymous and voluntary, and participants received no monetary compensation. Participation could be stopped at any time. The average survey completion time was 13.1 min (standard deviation [SD] = 4.1). This study was conducted according to the Declaration of Helsinki. The Ethics Committee of the Medical Faculty of the University of Duisburg-Essen approved the study (file number 20–9718-BO).

### Bariatric Surgery Procedures in Germany

In Germany, procedures regarding bariatric surgery are defined in S3 guidelines: surgery of obesity and metabolic diseases [27–29]. The guidelines specify aspects like patient selection, selection of the type of surgery, and perioperative management, as well as the structured follow-up. Regarding patient selection, the guidelines describe inclusion (e.g., BMI > 40 kg/m^2^ and conservative treatment options have been exhausted) as well as exclusion criteria (e.g., acute, untreated substance abuse or bulimia nervosa) which are reviewed by an inter-professional healthcare team prior to surgery and thus form the basis for selecting eligible patients for surgery. The decision for the type of bariatric surgery is made in consideration of the patients’ BMI, age, sex, comorbidities, adherence, profession, and preference as well as medical and psychosocial conditions. Before surgery, patients must undergo a medical and psychosocial assessment in which health-related, behavioral as well as laboratory parameters are considered. Moreover, patients are supported to improve pre-surgical success factors like weight loss and physical activity as well as through inpatient or outpatient psychotherapy. After bariatric surgery, patients have lifelong access to structured, inter-professional follow-up care. The guidelines describe the type and extent of aftercare (e.g., assessment of body weight and eating behavior, laboratory tests, and support to improve physical activity and take part in self-help groups).

### Measurements

The self-assessment consisted of several measurements regarding quality of care transitions during hospital discharge, patients’ characteristics, pre- and post-surgery body weight, and surgical intervention. Further, patient safety incidences were assessed.

We assessed the *quality of care transition* with the care transition measure (CTM) 30. This well-established 15-item scale measures patients’ evaluations of the hospital discharge process: their involvement in this process, obtained information about medications, subsequent medical examinations, and red flags they should observe to manage their health. Answers were provided on a 4-point Likert scale (1 = “strongly disagree’, 4 = “strongly agree’”). A fifth response option was “do not know/do not remember/not applicable,” which was not considered for the calculation of the CTM score. The CTM score was calculated as the unweighted average of all items. A sample item was “When I left the hospital, I had all the information I needed to be able to take care of myself.” Cronbach’s alpha was excellent with 0.95.

Regarding *patient safety incidents*, we asked participants whether they experienced any (1) unplanned readmissions to the hospital after their discharge from the hospital and if (2) medication complications occurred after bariatric surgery. Response options were “yes” vs. “no.”

We assessed various sociodemographic characteristics including age, sex, marital status, and educational degree. Furthermore, surgery-related (type of bariatric surgery and time since bariatric surgery) and nutrition-related information (general nutrition style, food intolerance) were captured.

To determine the weight results after bariatric surgery, we calculated the percentage of total weight loss (%TWL) as the preferred parameter for assessment of weight loss after bariatric surgery [31, 32]. For the calculation of %TWL, we used the following formula: %TWL = ((preoperative body weight – current body weight)/(preoperative body weight)) × 100 [32]. Weight regain is defined as a regain of body weight after an successful weight loss after bariatric surgery [6]. An overview of the questionnaire items is provided in Appendix 1.

### Statistical Analyses

We performed *t*-tests as well as analyses of variance (ANOVA) to determine mean group differences of %TWL for sociodemographic variables. Tukey multiple comparisons were subsequently used after ANOVA as post hoc tests to evaluate subgroup differences. We conducted stepwise linear regression models to investigate the relationship between the time since bariatric surgery and %TWL and the possible interacting effect of the quality of care transition. We used *R*^2^ to determine the amount of variance explanation of each model. Prior to this analysis, the CTM score was mean centered to be deployed as moderating factor. Moreover, to determine associations between quality of care transition and patient safety outcomes (i.e., “unplanned hospital readmissions” and “medication complications”), we performed binomial regression analyses. Patient safety outcomes were dummy coded (0 = experienced no hospital readmission/medication complications; 1 = experienced hospital readmission/medication complications). Odds ratio (OR) was used to estimate the outcome variables variance explained by quality of care transition. All statistical analyses were conducted in consideration of a significance level of *p* = 0.05 and performed using R and RStudio [33, 34].

## Results

### Sample Characteristics

To improve data quality, we excluded 38 participants based on an outlier analysis with regard to non-plausible completion time as well as a quality check of all items. The final sample used for data analyses consisted of *N* = 578 patients. Thereof, 435 female patients (75%) took part. Sex of patients was not related to %TWL (t[df = 483] = 0.09, *p* = 0.93). Mean age of patients in this study was M = 46.8 (SD = 10.0). Likewise, age was not associated to %TWL (*r* =  − 0.02, *p* = 0.65). Furthermore, our sample consisted of 284 patients who were married (49%), 97 who lived in partnership (17%), 82 who were single (14%), and 23 indicating other marital status (4%). Regarding education degree, 2 patients had no completed school degree (0%), 139 had a secondary school certificate (24%), 70 had a university entrance qualification (12%), 171 completed vocational training (30%), and 104 held a university’s degree (18%). Table 1 reports information on surgery- and nutrition-related characteristics. Further, difference tests regarding %TWL are included.

**Table 1:** Surgery and nutrition related characteristics and difference tests for the percentage of total weight loss (%TWL)

			%TWL		Test for difference (%TWL)	
Surgery and nutrition related characteristics	*N*	%	M	SD	Test statistic	*P* value
Type of bariatric surgery					*F*(3,471) = 3.05	0.03
Sleeve gastrectomy	321	56%	28.3	12.2		
Roux-en-Y gastric bypass	147	25%	32.0	12.6		
Omega loop bypass	65	11%	30.7	12.4		
Gastric band*	5	1%	41.0	20.8		
Single-anastomosis duodenal–ileal bypass with sleeve (SADI/S)*	6	1%	43.3	11.1		
Biliopancreatic division*	1	0%	33.1	-		
Other technique	32	6%	33.0	16.6		
Missing	1	0%	-	-		
Time since bariatric surgery					*F*(3,483) = 48.08	> 0.001
Less than 6 months	158	27%	17.8	7.8		
Less than 12 months	103	18%	33.0	7.5		
Less than 24 months	92	16%	38.6	9.9		
More than 24 months	224	39%	33.3	13.1		
Missing	1	0%	-	-		
General nutrition style┼					-	-
Omnivore diet	453	78%	29.8	12.8		
Vegetarian diet	16	3%	36.2	9.6		
Vegan diet	2	0%	36.2	4.1		
Other	17	3%	32.8	15.8		
Missing	90	16%	-	-		
Food intolerance					*t*(485) = − 3.36	> 0.001
No	399	69%	29.2	12.9		
Yes	89	15%	34.2	11.8		
Missing	90	16%	-	-		

^*^People who answered the assessment with this indication were excluded from the difference test due to small subgroup sample size. ┼No significance test was conducted due to small subgroup sample sizes

**Fig. 1:**
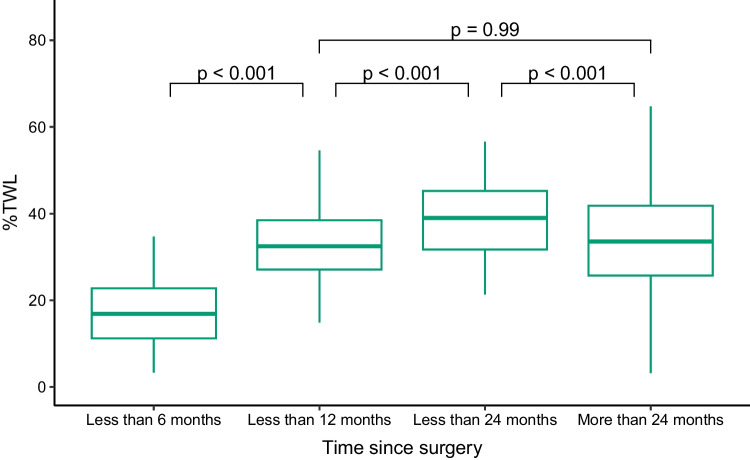
Percentage of total weight loss (%TWL) stratified for the time since bariatric surgery. Outliers are not displayed

To gain deeper understanding of subgroup differences, post hoc tests were performed. The Tukey multiple comparisons of means regarding the significant results of the type of bariatric surgery showed that patients with sleeve gastrectomy had a significantly lower %TWL than respondents with Roux-en-Y gastric bypass (*p* = 0.04). All other types of surgery did not significantly differ to each other. Further, post hoc comparisons regarding the significant results of time since bariatric surgery revealed that the %TWL significantly increased over time, until a time lag of “up to 24 months.” At this point, a significant decrease in %TWL was observed compared to patients who indicated to have received surgery more than 24 months ago. These results point out that a significant weight regain was present in our sample (cf. Fig. 1).

**Fig. 2:**
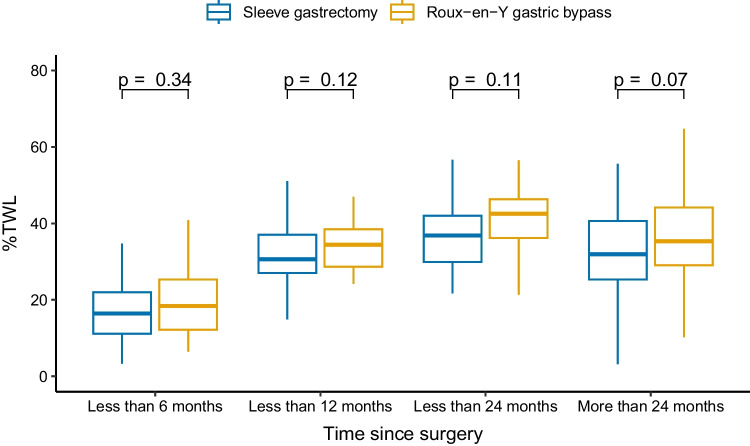
Percentage of total weight loss (%TWL) stratified for the time since bariatric surgery and the two most used surgical techniques (sleeve gastrectomy and Roux-en-Y gastric bypass). Outliers are not displayed

The two most used surgical techniques (*sleeve gastrectomy* and *Roux-en-Y gastric bypass*) showed an analogous trend in the variation of %TWL with no significant differences over the time since the surgical intervention (see Fig. 2).

The mean score of quality of care transition was 3.11 (SD = 0.68) and differed not significantly regarding the type of bariatric surgery (*F*[3,505] = 1.04, *p* = 0.38) or depending on the presence of a food intolerance (*t*[486] =  − 0.14, *p* = 0.89). Patients’ rating of the quality of care transition was significantly lower in patients who had their surgery more than 24 months ago than patients who had their surgery less than 12 or 6 months ago (*F*[3,517] = 5.48, *p* < 0.01).

### Influence of Quality of Care Transition on Association Between Time Since Surgery and %TWL

Given the above reported significant relationships between %TWL with *type of bariatric surgery* as well as with *food intolerances*, these two variables were included as covariates in multivariate analyses. Three stepwise regression models were conducted to determine the influence of quality of care transition on the association between time since surgery and %TWL. Results of this analyses are shown in Table 2.

**Table 2:** Stepwise regression analyses examining interaction effect of time since surgery and quality of care transition on %TWL

Predictor	Model 1	Model 2	Model 3
	Coefficient B	Coefficient B	Coefficient B
Time since surgery (TSS)	4.370**	-	4.466**
Quality of care transition (QCT)	-	0.434	− 4.642*
TSS x QCT	-	-	2.272**
Adjusted R^2^	20.9%	3.2%	23.6%

*N* = 475. * *p* < 0.05, ** *p* < 0.001, *R*^2^ variance explained

Covariates “type of bariatric surgery” and “food intolerances” were significant in each model. Time since surgery was treated as continuous variable to facilitate interpretation of results

*Model 1*, %TWL was regressed on time since surgery and covariates

*Model 2*, %TWL was regressed on quality of care transition and covariates

*Model 3*, model 1 + model 2 + interaction (i.e., multiplicative term) of time since surgery and quality of care transition

**Fig. 3:**
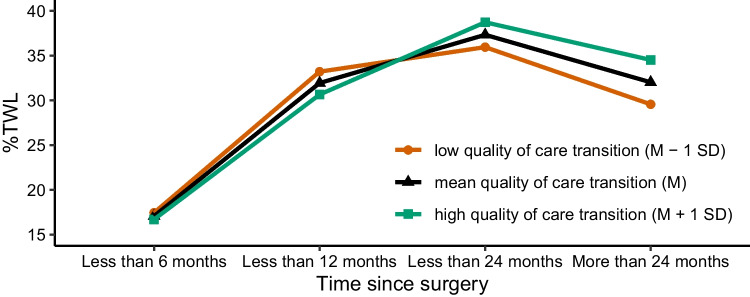
Significant interaction effect of time since surgery and the quality of care transition on %TWL. The illustrated values were adjusted for the covariates “type of bariatric surgery” and “food intolerances”

The results of model 1 confirm that %TWL is significantly related to the time since surgery. Model 2 reveals that %TWL is not significantly related to the quality of care transition. However, model 3 shows that there is a significant interaction effect of time since surgery and quality of care transition (i.e., with a *R*^2^ change of 2.7%). These results suggest that the quality of care transition moderated the effect of time since surgery on %TWL such as that patients who experienced a better transition of care process during hospital discharge reported a higher %TWL after 24 months. This relationship is shown in Fig. 3.

As Fig. 3 shows, participants who reported a high quality of care transition (green line, M + 1 SD) also experienced a weight gain when the surgery was more than 24 months ago. Nevertheless, the weight regain was significantly lower compared to patients who experienced low quality of care transitions.

### Analyses of Quality of Care Transition and Patient Safety Incidents

In our sample, 70 out of 498 individuals had an unplanned readmission to the hospital after their surgery, resulting in an incidence of 14.1%. Medication complications after discharge were experienced by 60 out of 498 participants, indicating an incidence of 12.1%. As time since surgery is presumably an important influence whether individuals experience medication complications or hospital readmissions, we considered this variable as a covariate in the following analyses. The results of binomial regression analyses on unplanned readmission and medication complications are presented in Table 3.

**Table 3:** Binomial regression analyses examining the effect of the quality of care transition on unplanned hospital readmissions and experienced medication complications

	Patient safety incidents	
Predictor	Hospital readmission	Medication complication
	Coefficient B (OR)	Coefficient B (OR)
Time since surgery	0.265* (1.30)	0.001 (1.00)
Quality of care transition	− 0.399* (0.67)	− 0.744** (0.48)

*N* = 498. * *p* < 0.05, ** *p* < 0.001. Time since surgery was considered as continuous variable to facilitate interpretation of results

Coding: 0 = experienced no hospital readmission/medication complications; 1 = experienced hospital readmission / medication complications

Results in Table 3 reveal that the quality of care transition is associated with both less unplanned hospital readmissions (OR = 0.67; 95% confidence interval [CI], 0.47–0.96) and fewer medication complications after bariatric surgery (OR = 0.48; 95% CI, 0.32–0.69) when controlled for the time since surgery.

## Discussion

Our results corroborate that the quality of care transition during the hospital discharge process is a crucial success factor for medium-term success and mitigation of patient safety incidents in bariatric surgical care. Specifically, our results underline that weight regain is a challenge for patients after bariatric interventions. Nevertheless, we showed that weight regain is mitigated when the quality of care transition during hospital discharge is high. Moreover, our findings show that patient safety incidents were more likely when the quality of care transition was low.

Regarding our first question, our findings emphasize the utter relevance of quality of care transitions during bariatric patients’ discharge from the hospital: patients who reported high quality of care transitions had significantly lower weight regain 24 months after surgery compared to patients who reported average or low quality of care transition in the discharge process. High quality of care transitions safeguard effective and smooth hospital discharge of patients and ensure complete patient and information transfer across different providers. We further assume that a higher quality of care transition contributes to increased patient empowerment. High empowerment leads to greater compliance with providers’ behavioral recommendations (e.g., nutrition recommendations) after bariatric surgery. This finding resonates well with previous research on patient empowerment, compliance, and adherence to health-beneficial behaviors in the context of medication adherence [35], adherence to hand hygiene [36], and also in long-term weight maintenance [37]. In the context of bariatric surgery, our observations suggest that the positive effect of high quality of care transitions does not show up in the first months after surgery but reveals after approximately 24 months with patients being affected by a substantial weight regain. This could be explained by the fact that initially all patients benefit from bariatric surgery, regardless of the quality of care transition. However, successful weight loss and long-term weight maintenance are dependent on high compliance and adherence with post-surgical behavior recommendations [38–40]. Our results corroborate that successful compliance and adherence are promoted by high quality of care transitions that in turn are associated with a higher likelihood of medium-term weight maintenance. Future studies should investigate the relationships between the quality of discharge and care transition, patient engagement in recognition of early complications and readmissions, as well as medium- to long-term weight loss. Future studies should therefore apply prospective designs to verify our assumptions in one comprehensive model as well as test for different behavioral outcomes across different time lags, i.e., effects of quality of care transitions in short-term recognition of surgical complications and readmission compared to medium- and long-term weight regain.

Concerning our second question on adverse patient safety incidents, our findings show that high quality of care transitions was associated with lower likelihood of unplanned hospital readmission as well as fewer medication complications at home after bariatric surgery. Both results highlight that measures to foster patient safety are not limited to the actual intervention and care in the hospital but also play an important role in the discharge process. Despite the existing research base on possible contributing factors of readmissions after bariatric surgery [41–43], to the best of our knowledge, there is no study investigating potential mitigating factors within the discharge process. Frequently, the discharge process is the last point of contact of patients with the operating hospitals’ healthcare professionals. Our findings suggest that missing information and low patient centeredness during this transition are potentially associated with uncertainty among patients, which in turn results in reduced self-efficacy, lower compliance with post-surgical behavior recommendations, and more hospital readmissions [44, 45].

Altogether, our results emphasize the sustaining importance of high quality of care transitions after bariatric surgery. High quality of care transitions during patients’ discharge are characterized by high levels of patient centeredness and comprehensive communication of relevant information to support patients in assuming responsibility. Our findings underline that effective patient discharge processes support the medium-term success of bariatric surgery and prevent the occurrence of adverse patient safety incidents at home. Hospitals and healthcare professionals should invest resources into the discharge process to foster patients’ engagement and their compliance to behavioral recommendations with the aim to prevent weight regain as well as patient safety incidents after bariatric surgery. Moreover, health policy decisions should be made to invigorate the discharge process as an effective driver of patient empowerment. Research has shown that several determinants have an impact on the sustained success of bariatric surgery. Our study provides first evidence for the importance of the quality of discharge processes in supporting the patients after surgery. Future research as well as practitioners could consider this evidence to further develop the knowledge and procedures of bariatric surgery.

This study is subject to some limitations. First, the results of our study cannot be interpreted in a causal meaning as we utilized a cross-sectional study design. Our sample consisted of heterogeneous participants regarding the time since their bariatric surgery. On the one hand, this is a strength of our study as we could examine and compare patients with different time lags since bariatric surgery. On the other hand, memory biases regarding the quality of patients’ care transition may have occurred. Different recall or hindsight biases as well as retrospective appraisals may occur across different time lags after surgery that were considered in this study. We included several hospitals in our sample as we used an online flyer containing information on the study to recruit participants in topic-related social media groups what strengthens the external validity. Nevertheless, the study sample was partly recruited in one hospital which could facilitate a sampling bias. Longitudinal studies are needed to examine causal effects between the quality of care transition and health-related outcomes and patient safety incidents across various hospitals and surgical facilities with potentially varying discharge practices (e.g., accredited obesity outpatient care facilities or patient support programs after bariatric surgery). We did not distinguish between primary and revisional surgery in our study; however, future studies should consider this potentially relevant factor when assessing the relevance of quality of care transition after bariatric surgery. In addition, we did not capture adherence or compliance directly. Thus, future studies should observe compliance to post-bariatric surgery nutrition recommendations [46] or micronutrient supplementation behavior [47]. Furthermore, future studies should consider possible mediating variables to shed light on the influence of the quality of care transition on the engagement and empowerment of patients after bariatric surgery. Especially, prospective studies should be conducted to replicate our findings as well as to investigate causal relations within individual patient journeys.

## Conclusion

The quality of care transitions after bariatric surgery was associated with weight gain as well as patient safety. Hospitals should therefore design effective and patient-centered discharge processes to meet patient requirements and promote compliance. Our study may inform practice interventions on patient-centered care transitions that promote adherence to post-surgical behavior recommendations with notable effects on weight maintenance and mitigation of adverse patient safety incidents at home.

## Data Availability

The datasets generated during and/or analysed during the current study are available from the corresponding author on reasonable request.

## Appendix 1. Questionnaire items

Quality of care transition (CTM; ©Eric A. Coleman, MD, MPH, all rights reserved)

Response options: 1, strongly disagree; 4, strongly agree; 5, do not know/do not remember/not applicable.

**Table:**

#	Item
1	Before I left the hospital, the staff and I agreed about clear health goals for me and how these would be reached
2	The hospital staff took my preferences and those of my family or caregiver into account in deciding what my health care needs would be when I left the hospital
3	The hospital staff took my preferences and those of my family or caregiver into account in deciding where my health care needs would be met when I left the hospital
4	When I left the hospital, I had all the information I needed to be able to take care of myself
5	When I left the hospital, I clearly understood how to manage my health
6	When I left the hospital, I clearly understood the warning signs and symptoms I should watch for to monitor my health condition
7	When I left the hospital, I had a readable and easily understood written plan that described how all of my health care needs were going to be met
8	When I left the hospital, I had a good understanding of my health condition and what makes it better or worse
9	When I left the hospital, I had a good understanding of the things I was responsible for in managing my health
10	When I left the hospital, I was confident that I knew what to do to manage my health
11	When I left the hospital, I was confident I could actually do the things I needed to do to take care of my health
12	When I left the hospital, I had a readable and easily understood written list of the appointments or tests I needed to complete within the next several weeks
13	When I left the hospital, I clearly understood the purpose for taking each of my medications
14	When I left the hospital, I clearly understood how to take each of my medications, including how much I should take and when
15	When I left the hospital, I clearly understood the possible side effects of each of my medications

## Patient safety incidents

Response options: yes vs. no.

**Table:**

Variable	Item
Unplanned hospital readmissions	Did you need to be readmitted to a hospital/emergency room unplanned after your discharge from the hospital?
Medication complications	Have you had any difficulties with taking your medication (e.g., wrong dosage; unexpected side effects)?

## Sociodemographic characteristics

**Table:**

Variable	Item	Response options
Age	Your age	Text box
Sex	Your gender	1 = female 2 = male 3 = diverse
Marital status	Your marital status	1 = married 2 = not married, but in partnership 3 = single 4 = other
Educational degree	Your highest educational degree attained	1 = no school degree 2 = secondary school certificate (Hauptschule) 3 = secondary school certificate (Mittlere Reife) 4 = university entrance qualification (Fachhochschulreife) 5 = university entrance qualification (Allgemeine Hochschulreife) 6 = completed vocational training 7 = university’s degree (Fachhochschule) 8 = university’s degree (Universität)
Preoperative body weight	What was your body weight before surgery?	Text box
Postoperative body weight	What is your current body weight?	Text box
Body height	Your height in centimeters:	Text box

## Surgery-related information

**Table:**

Variable	Item	Response options
Type of bariatric surgery	What technique of gastric surgery or gastrointestinal surgery was applied to you?	1 = sleeve gastrectomy 2 = Roux-en-Y gastric bypass 3 = omega loop bypass 4 = gastric band 5 = single anastomosis 6 = duodenal–ileal bypass with sleeve (SADI/S) 7 = biliopancreatic division 8 = other technique
Time since bariatric surgery	How long ago was the operation?	1 = less than 6 months 2 = less than 12 months 3 = less than 24 months 4 = more than 24 months

## Nutrition-related information

**Table:**

Variable	Item	Response options
General nutrition style	How do you eat in general?	1 = omnivore 2 = vegetarian 3 = vegan 4 = other
Food intolerance	Do you have a food allergy or food intolerance?	1 = no 2 = yes

## References

[1] Velapati SR, Shah M, Kuchkuntla AR, Abu-dayyeh B, Grothe K, Hurt RT (2018). Weight regain after bariatric surgery: prevalence, etiology, and treatment. Curr Nutr Rep.

[2] Voorwinde V, Hoekstra T, Monpellier VM, Steenhuis IHM, Janssen IMC, van Stralen MM (2022). Five-year weight loss, physical activity, and eating style trajectories after bariatric surgery. Surg Obes Relat Dis.

[3] Magro DO, Geloneze B, Delfini R, Pareja BC, Callejas F, Pareja JC (2008). Long-term weight regain after gastric bypass: a 5-year prospective study. Obes Surg.

[4] Dimeglio C, Becouarn G, Topart P, Bodin R, Buisson JC, Ritz P (2020). Weight loss trajectories after bariatric surgery for obesity: mathematical model and proof-of-concept study. JMIR Med Inform.

[5] Istfan NW, Lipartia M, Anderson WA, Hess DT, Apovian CM (2021). Approach to the patient: management of the post–bariatric surgery patient with weight regain. J Clin Endocrinol Metab.

[6] El Ansari W, Elhag W (2021). Weight regain and insufficient weight loss after bariatric surgery: definitions, prevalence, mechanisms, predictors, prevention and management strategies, and knowledge gaps—a scoping review. Obes Surg.

[7] Athanasiadis DI, Martin A, Kapsampelis P, Monfared S, Stefanidis D (2021). Factors associated with weight regain post-bariatric surgery: a systematic review. Surg Endosc.

[8] Yarigholi F, Bahardoust M, Mosavari H, Tehrani FM, Gholizadeh H, Shahmiri SS, et al. Predictors of weight regain and insufficient weight loss according to different definitions after sleeve gastrectomy: a retrospective analytical study. Obes Surg. 2022 [cited 2022 Oct 20]. 10.1007/s11695-022-06322-3 10.1007/s11695-022-06322-3 36260221

[9] Santos C, Carvalho M, Oliveira L, Palmeira A, Rodrigues LM, Gregório J (2022). The long-term association between physical activity and weight regain, metabolic risk factors, quality of life and sleep after bariatric surgery. Int J Environ Res Public Health.

[10] Egberts K, Brown WA, Brennan L, O’Brien PE (2012). Does exercise improve weight loss after bariatric surgery? A systematic review. Obes Surg.

[11] Cornejo-Pareja I, Molina-Vega M, Gómez-Pérez AM, Damas-Fuentes M, Tinahones FJ (2021). Factors related to weight loss maintenance in the medium–long term after bariatric surgery: a review. J Clin Med.

[12] Tolvanen L, Christenson A, Surkan PJ, Lagerros YT (2022). Patients’ experiences of weight regain after bariatric surgery. Obes Surg.

[13] Cambi MPC, Baretta GAP, Magro DDO, Boguszewski CL, Ribeiro IB, Jirapinyo P (2021). Multidisciplinary approach for weight regain—how to manage this challenging condition: an expert review. Obes Surg.

[14] Belligoli A, Bettini S, Segato G, Busetto L (2020). Predicting responses to bariatric and metabolic surgery. Curr Obes Rep.

[15] Cao X, Chen L, Diao Y, Tian L, Liu W, Jiang X. Validity and reliability of the Chinese version of the care transition measure. Christiansen H, editor. PLOS One. 2015;10:e0127403. 10.1371/journal.pone.0127403 PMC4441382 26000708

[16] Shadmi E, Zisberg A, Coleman EA (2009). Translation and validation of the care transition measure into Hebrew and Arabic. Int J Qual Health Care.

[17] Bakshi AB, Wee S-L, Tay C, Wong L-M, Leong IY-O, Merchant RA (2012). Validation of the care transition measure in multi-ethnic South-East Asia in Singapore. BMC Health Serv Res.

[18] Coleman EA, Smith JD, Frank JC, Eilertsen TB, Thiare JN, Kramer AM (2002). Development and testing of a measure designed to assess the quality of care transitions. Int J Integr Care.

[19] Flink M, Ekstedt M (2017). Planning for the discharge, not for patient self-management at home – an observational and interview study of hospital discharge. Int J Integr Care.

[20] Elwyn G, Nelson E, Hager A, Price A (2020). Coproduction: when users define quality. BMJ Qual Saf.

[21] Vincent CA, Coulter A (2002). Patient safety: what about the patient?. Qual Saf Health Care.

[22] Náfrádi L, Nakamoto K, Schulz PJ. Is patient empowerment the key to promote adherence? A systematic review of the relationship between self-efficacy, health locus of control and medication adherence. PLOS One. Public Library of Science; 2017;12:e0186458. 10.1371/journal.pone.0186458 PMC5645121 29040335

[23] Manafo E, Petermann L, Mason-Lai P, Vandall-Walker V (2018). Patient engagement in Canada: a scoping review of the ‘how’ and ‘what’ of patient engagement in health research. Health Res Policy Syst.

[24] Orozco-Beltrán D, Morales C, Artola-Menéndez S, Brotons C, Carrascosa S, González C (2022). Effects of a digital patient empowerment and communication tool on metabolic control in people with type 2 diabetes: the DeMpower Multicenter Ambispective Study. JMIR Diabetes.

[25] Mishali M, Kisner M (2022). Psycho-behavioral factors related to weight regain after bariatric surgery. Obes Surg.

[26] Ugarte C, Quiñones Á, Saúl LA (2022). Relationship among self-efficacy expectations, locus of control, and attributions in bariatric patients. Int J Environ Res Public Health.

[27] Deutsche Gesellschaft für Allgemein- und Viszeralchirurgie. S3-Leitlinie Chirurgie der Adipositas und metabolischer Erkrankungen [Internet]. [cited 2022 Dec 29]. Available from: https://register.awmf.org/assets/guidelines/088-001l_S3_Chirurgie-Adipositas-metabolische-Erkrankugen_2018-02.pdf

[28] Runkel N, Colombo-Benkmann M, Hüttl TP, Tigges H, Mann O, Flade-Kuthe R (2011). Evidence-based German guidelines for surgery for obesity. Int J Colorectal Dis.

[29] Dietrich A, Aberle J, Wirth A, Müller-Stich B, Schütz T, Tigges H (2018). Obesity surgery and the treatment of metabolic diseases. Dtsch Ärztebl Int.

[30] Coleman EA, Mahoney E, Parry C (2005). Assessing the quality of preparation for posthospital care from the patient’s perspective: the care transitions measure. Med Care Lippincott Williams Wilkins.

[31] Grover BT, Morell MC, Kothari SN, Borgert AJ, Kallies KJ, Baker MT (2019). Defining weight loss after bariatric surgery: a call for standardization. Obes Surg.

[32] Sabench Pereferrer F, Molina López A, Vives Espelta M, Raga Carceller E, Blanco Blasco S, Buils Vilalta F (2017). Weight loss analysis according to different formulas after sleeve gastrectomy with or without antral preservation: a randomised study. Obes Surg.

[33] R Core Team. R: A language and environment for statistical computing. R Foundation for Statistical Computing [Internet]. Vienna, Austria; 2021. https://www.r-project.org/

[34] RStudio Team. RStudio: Integrated Development for R [Internet]. Boston, MA: RStudio, PBC; 2020. http://www.rstudio.com/

[35] Pileggi C, Caligiuri E, Nobile CGA, Pavia M (2018). Information about management of chronic drug therapies prescribed at hospital discharge: does it affect patients’ knowledge and self-confidence?. BMC Health Serv Res.

[36] Görig T, Dittmann K, Kramer A, Heidecke C-D, Diedrich S, Hübner N-O (2019). Active involvement of patients and relatives improves subjective adherence to hygienic measures, especially selfreported hand hygiene: results of the AHOI pilot study. Antimicrob Resist Infect Control.

[37] Bjerkan KK, Sandvik J, Nymo S, Græslie H, Johnsen G, Mårvik R (2022). The long-term impact of postoperative educational programs on weight loss after Roux-en-Y gastric bypass. Obes Surg.

[38] Sarwer DB, Dilks RJ, West-Smith L (2011). Dietary intake and eating behavior after bariatric surgery: threats to weight loss maintenance and strategies for success. Surg Obes Relat Dis.

[39] Masood A, Alsheddi L, Alfayadh L, Bukhari B, Elawad R, Alfadda AA. Dietary and lifestyle factors serve as predictors of successful weight loss maintenance postbariatric surgery. J Obes. Hindawi; 2019;2019:e7295978. 10.1155/2019/7295978 PMC6390255 30891313

[40] Herman KM, Carver TE, Christou NV, Andersen RE (2014). Keeping the weight off: physical activity, sitting time, and weight loss maintenance in bariatric surgery patients 2 to 16 years postsurgery. Obes Surg.

[41] Al-Mazrou AM, Cruz MV, Dakin G, Bellorin O, Pomp A, Afaneh C (2021). Stratification of readmission after bariatric surgery by day of post-discharge presentation. Obes Surg.

[42] Dreifuss NH, Xie J, Schlottmann F, Cubisino A, Baz C, Vanetta C (2022). Risk factors for readmission after same-day discharge sleeve gastrectomy: a metabolic and bariatric surgery accreditation and quality improvement program database analysis. Obes Surg.

[43] Reyes-Pérez A, Sánchez-Aguilar H, Velázquez-Fernández D, Rodríguez-Ortíz D, Mosti M, Herrera MF (2016). Analysis of causes and risk factors for hospital readmission after Roux-en-Y gastric Bypass. Obes Surg.

[44] Backman C, Chartrand J, Crick M, Devey Burry R, Dingwall O, Shea B (2021). Effectiveness of person- and family-centred care transition interventions on patient- oriented outcomes: a systematic review. Nurs Open.

[45] Hume AL, Kirwin J, Bieber HL, Couchenour RL, Hall DL, Pharmacy AC of C (2012). Improving care transitions: current practice and future opportunities for pharmacists. Pharmacother J Hum Pharmacol Drug Ther.

[46] Bäuerle A, Schräpler L, Marsall M, Engelmann G, Knoll-Pientka N, Schüren LC, et al. Development and validation of dietary behavior inventory—surgery (DBI-S) in the scope of international post-bariatric surgery guidelines and recommendations. Nutrients. Multidisciplinary Digital Publishing Institute; 2022;14:3692. 10.3390/nu14183692 PMC9504912 36145070

[47] Mahawar KK, Clare K, O’Kane M, Graham Y, Callejas-Diaz L, Carr WRJ (2019). Patient perspectives on adherence with micronutrient supplementation after bariatric surgery. Obes Surg.

